# Identification of the Pangenome and Its Components in 14 Distinct *Aggregatibacter actinomycetemcomitans* Strains by Comparative Genomic Analysis

**DOI:** 10.1371/journal.pone.0022420

**Published:** 2011-07-19

**Authors:** Weerayuth Kittichotirat, Roger E. Bumgarner, Sirkka Asikainen, Casey Chen

**Affiliations:** 1 Department of Microbiology, University of Washington, Seattle, Washington, United States of America; 2 Department of Surgical Sciences, Periodontology, Kuwait University, Kuwait City, Kuwait; 3 Division of Periodontology, Diagnostic Sciences and Dental Hygiene, Herman Ostrow School of Dentistry of the University of Southern California, Los Angeles, California, United States of America; Baylor College of Medicine, United States of America

## Abstract

**Background:**

*Aggregatibacter actinomycetemcomitans* is genetically heterogeneous and comprises distinct clonal lineages that may have different virulence potentials. However, limited information of the strain-to-strain genomic variations is available.

**Methodology/Principal Findings:**

The genome sequences of 11 *A. actinomycetemcomitans* strains (serotypes a-f) were generated *de novo*, annotated and combined with three previously sequenced genomes (serotypes a-c) for comparative genomic analysis. Two major groups were identified; serotypes a, d, e, and f, and serotypes b and c. A serotype e strain was found to be distinct from both groups. The size of the pangenome was 3,301 genes, which included 2,034 core genes and 1,267 flexible genes. The number of core genes is estimated to stabilize at 2,060, while the size of the pangenome is estimated to increase by 16 genes with every additional strain sequenced in the future. Within each strain 16.7–29.4% of the genome belonged to the flexible gene pool. Between any two strains 0.4–19.5% of the genomes were different. The genomic differences were occasionally greater for strains of the same serotypes than strains of different serotypes. Furthermore, 171 genomic islands were identified. Cumulatively, 777 strain-specific genes were found on these islands and represented 61% of the flexible gene pool.

**Conclusions/Significance:**

Substantial genomic differences were detected among *A. actinomycetemcomitans* strains. Genomic islands account for more than half of the flexible genes. The phenotype and virulence of *A. actinomycetemcomitans* may not be defined by any single strain. Moreover, the genomic variation within each clonal lineage of *A. actinomycetemcomitans* (as defined by serotype grouping) may be greater than between clonal lineages. The large genomic data set in this study will be useful to further examine the molecular basis of variable virulence among *A. actinomycetemcomitans* strains.

## Introduction

Gram-negative *Aggregatibacter actinomycetemcomitans* is assumed to be the primary etiologic agent of localized aggressive periodontitis [1] and has also been implicated in chronic periodontitis and severe non-oral infections [2]. The bacterium has a complex lifecycle. It is acquired through transmission from infected individuals [3], [4], and may initially colonize oral mucosa possibly as a facultative intracellular pathogen [5], [6]. The bacterium moves from the initial oral colonization site to subgingival crevices and competes with other bacteria in the niche. Successful establishment of persistent colonization in subgingival crevices by *A. actinomycetemcomitans* may lead to periodontal destruction and development of periodontitis in susceptible individuals [7], [8]. To initiate a new cycle, the bacterium is transmitted by saliva to a new host [4].

The virulence potential of *A. actinomycetemcomitans* appears to vary among strains. Specific serotypes/clonal types of *A. actinomycetemcomitans* have been reported to be over-represented in periodontitis [9], [10]. Subjects infected by strains of serotype b JP2 clone (defined by a 530-bp deletion in the promoter region of the leukotoxin operon) were shown to be at a higher risk of exhibiting progressing periodontal disease or developing aggressive periodontitis than by strains of “non-JP2 clones” [7], [11]. The within-species variable virulence may be attributed to strain-to-strain variation in genome content and regulation of virulence gene expressions [12], [13]. Comparative genomics of *A. actinomycetemcomitans* strains may yield insight to the molecular basis of such variation.

The complete genome sequence of *A. actinomycetemcomitans* serotype b strain HK1651 has been available since 2002 (http://www.genome.ou.edu/act.html). The genome sequences of a serotype a strain D7S-1 and serotype c strain D11S-1 were recently published by our laboratory [14], [15]. In the present study, the genome sequences of an additional 11 *A. actinomycetemcomitans* strains were generated *de novo*, and combined with the three available genome sequences for comparative genomic analysis. The protein-coding genes of the genomes were compared among strains to determine the size of the core and flexible gene pools, the pangenome, the gain/loss of putative virulence determinants, and to identify genomic islands within each strain. The results demonstrated substantial differences in genomic content that included functionally undefined genes, well-established virulence determinants and newly identified genomic islands among the 14 *A. actinomycetemcomitans* strains.

## Results and Discussion

### Genome sequencing, gene finding and annotation

Table 1 provides a summary of the 11 strains sequenced in this study as well as the three previously sequenced strains of *A. actinomycetemcomitans*. These strains were recovered from the subgingival plaque of non-cohabiting individuals with different periodontal disease diagnoses (periodontally healthy, gingivitis, chronic periodontitis, and aggressive periodontitis), and included a strain of JP2 clone (HK1651).

**Table 1:** Summary information of *A. actinomycetemcomitans* strains.

Strain	D7S-1	D17P-3	H5P1	HK1651	ANH9381	I23C	SCC1398	SCC2302	D11S-1	D17P-2	I63B	SCC393	SC1083	D18P-1
**Age and ethnicity of the subject**	29African-American	24Asian-American	27Asian-American	18African (Ghana)	>50Caucasian	48Caucasian	25Caucasian	33Caucasian	16African-American	24Asian-American	49Caucasian	40Caucasian	unknown	20Asian-American
**Geographic location**	USA	USA	USA	Ghana	Finland	Finland	Finland	Finland	USA	USA	Finland	Finland	USA	USA
**Serotype** a	a	a	a	b	b	b	b	c	c	c	d	e	e	f
**Diagnosis** b	GAP	LAP	H	LAP or GAP^c^	H	CP, mild	LAP	G	GAP	LAP	H	CP, severe	Unknown	GAP
**Genome size (bp)**	2308328	2384518^d^	2151174^d^	2105503	2112345^d^	2020423^d^	2167288^d^	2037488^d^	2105764	2173675^d^	2175418^d^	2222051^d^	2164541^d^	2237538^d^
**% G+C**	44.3	43.9	44.5	44.4	44.4	44.9	44.5	44.4	44.6	44.3	44.3	44.4	44.6	44.1
**No. of rRNA genes** e	19	NA	NA	19	NA	NA	NA	NA	19	NA	NA	NA	NA	NA
**No. of tRNA genes**	53	49	33	55	44	26	39	44	55	47	40	35	46	46
**No. of protein-coding gene clusters**	2581	2880	2546	2444	2497	2473	2437	2442	2656	2611	2558	2669	2505	2614
**No. of genomic islands**	26	19	5	8	9	4	9	10	14	13	10	15	11	18
**Size range of islands (bp)**	5046–22209	5046–26606	5037–11447	5040–23912	5046–23911	5682–6844	5047–23913	5043–20734	5047–43655	5046–31797	5046–16499	5078–40031	5647–23887	5047–20157
**Median size of islands (bp)**	8910	9368	5487	10007	7862	6197	8307	6707	7345	7528	7502	6981	7905	10148
**%GC of islands**	31–50	30–50	37–50	32–50	31–50	36–49	32–50	34–47	33–50	33–47	31–50	29–50	34–48	28–47
**Genome signature of islands** f	90–235	118–276	122–226	99–201	110–232	134–192	136–201	131–206	132–210	133–213	95–251	112–320	124–203	99–266
**Codon bias of islands** f	340–636	311–836	395–647	318–577	337–591	415–626	440–550	408–592	407–581	434–591	356–655	373–659	369–588	307–733

a Serotype was determined by PCR analysis supported by the presence of the serotype-specific gene cluster in the genome.

b GAP, generalized aggressive periodontitis; LAP, localized aggressive periodontitis; H, periodontally healthy; CP, chronic periodontitis; G, chronic gingivitis.

c Based on the information presented at ATCC for strain HK1651.

d Estimated based on the total number of bases in the large contigs.

e NA, not available in the draft genomes due to repetition of the same gene sequences in the genomes.

f For definitions of genome signature and codon bias see [28].

The % G+C of the genomes is in the range of 43.9–44.9 as expected for the species. Individual genomes varied in size from 2.02 to 2.38 Mb (mean±S.D: 2.17±0.12 Mb). Excluding RNA genes, individual genomes contained 2,442–2,880 protein-coding genes. The numbers of protein-coding genes represent an upper limit since they were identified by a combination of different approaches and included genes that were relatively short (100–200 bp). Also, the results included genes that may belong to plasmids or phages. Overall, the genomic variations in size or the numbers of protein-coding genes were <20% between any two strains.

The sequence quality of the 11 *A. actinomycetemcomitans* genomes was high (see Table S1 for detail of sequence quality). The depths of the genome sequencing were 16× to 43×. The percentage of Q39 (ie, the percentage of bases with a quality score of less than or equal to 39 which is equivalent to an error rate of approximately 1 in 10,000 bases) was less than 1% in 11 of the 12 genomes, and only 1.6% in the remaining genome.

A total of 33,626 predicted genes were found in the genomes of 14 *A. actinomycetemcomitans* strains and grouped into 3,338 homologous gene clusters. Gene members within each homologous gene cluster showed at least 75% DNA sequence similarity to the cluster representative sequence (the longest member) suggesting meaningful homologous gene grouping result (see Figure S1). The homologous gene clusters data used in this study can be accessed by using our online tool, which can be found at http://expression.washington.edu/genetable/script/gene_table_viewer. A more detailed description of our homologous gene grouping method as well as our web tool for browsing through the homologous gene cluster data will be presented in a separate paper.

Two approaches were employed in this study to overcome possible sequencing errors, missed sequences and fragmented genes expected in the draft genomes. First, the gene clusters were analyzed instead of the individual genes. By using this approach the split genes were recognized and assigned to the same gene clusters. Second, the representative gene sequences of each gene cluster were used to search for homologs in the genomes. To validate our approaches, we analyzed the gene prediction results using the complete genomes of strains D7S-1 and D11S-1 and the unfinished genomes (ie, contigs derived from 454 sequencing) of the strains. Using the unfinished genomes would have missed a total of three out of 5,237 genes between these strains (Table 2). Our approaches also avoided common sequencing errors involving polynucleotides. As an example we identified a sequencing error in *ltxC* of strain D7S-1, which missed a “T” at the end of a poly-T track at the nucleotide coordinates 601,187–601,190 (sequencing data not shown). Importantly, the gene was correctly identified in spite of the sequencing error.

**Table 2:** Comparison of gene identification in complete and draft genomes of *A. actinomycetemcomitans* strains D7S-1 and D11S-1.

Strains	D7S-1	D11S-1
Draft genome total length (bp)*	2,203,297	2,169,704
Complete genome total length (bp)	2,308,328	2,222,842
Total protein-coding genes in draft genome	2,579	2,655
Total protein-coding genes in complete genome	2,581	2,656

*106 large contigs for D7S-1 and 199 large contigs for D11S-1.

### Phylogenetic analysis of *A. actinomycetemcomitans* and closely related species

**Figure 1:**
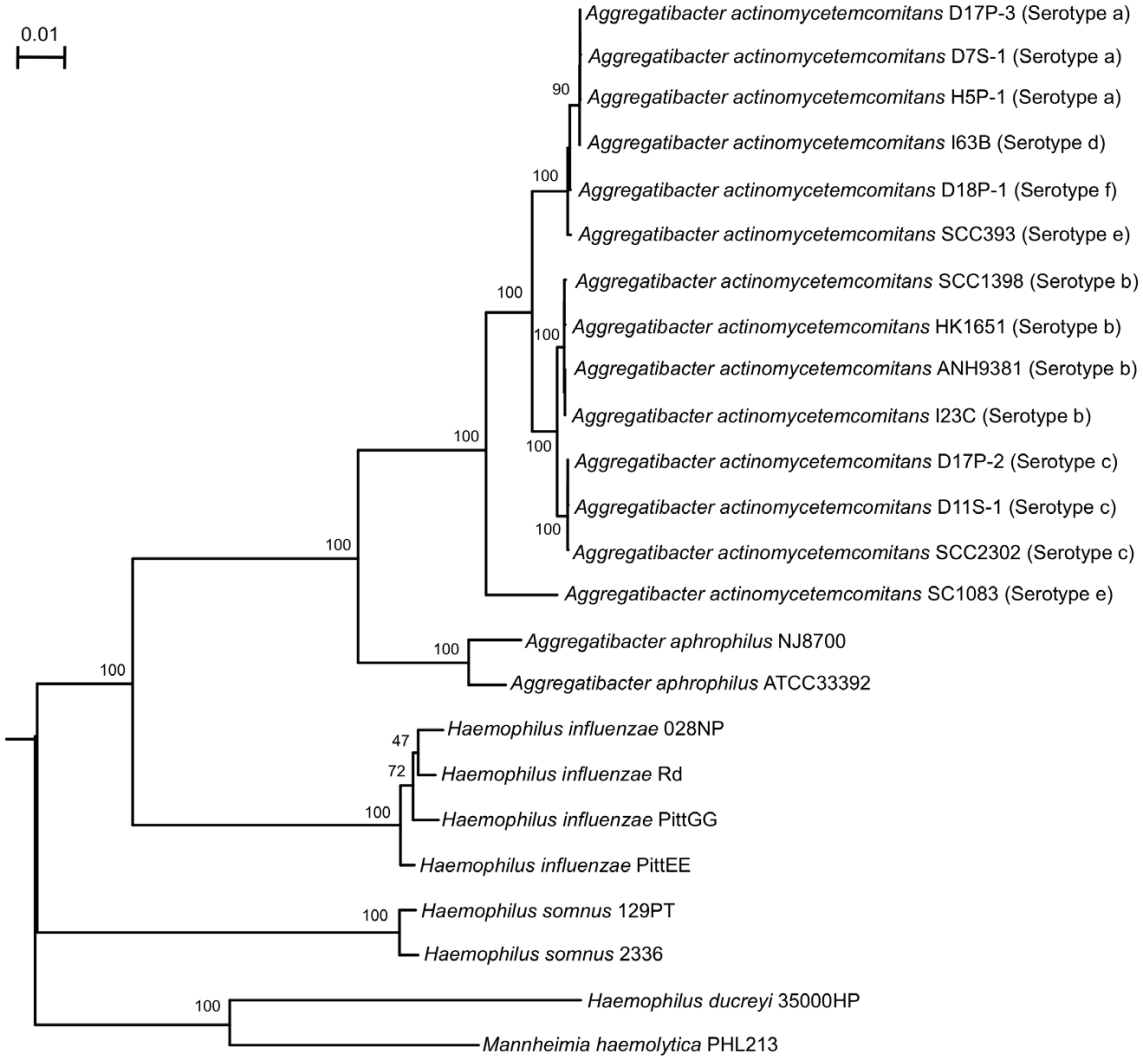
Phylogenetic tree of *A. actinomycetemcomitans* and related species based on housekeeping genes. The phylogenetic relationships of *A. actinomycetemcomitans*, *A. aphrophilus*, *Haemophilus* spp. and *M. haemolytica* strains were examined with 25 housekeeping gene (a total length of 17,840 bp) sequences. Bootstrap values (100 replicates) are given at branch points. Bar represents substitutions per site. *M. haemolytica* strain PHL213 was used to root the tree. Serotype information is given for all *A. actinomycetemcomitans* strains. The phylogenetic tree was constructed and drawn using PHYLIP version 3.6.

The sequences of 25 housekeeping genes (a total length of 17,840 bp) (Table S2) common to *Aggregatibacter actinomycetemcomitans* and related species (*Aggregatibacter aphrophilus*, *Haemophilus influenzae*, *Haemophilus somnus*, *Haemophilus ducreyi* 35000HP, and *Mannheimia haemolytica*) were used for phylogenetic analysis. The results are shown in Figure 1. Strains of serotypes a, d, e and f (except serotype e strain SC1083) formed a group, while serotypes b and c strains formed a separate group. The serotype e strain SC1083 appeared to be equally distinct from the two major *A. actinomycetemcomitans* groups. To the best of our knowledge, this is the first study that used the sequences of a large number of housekeeping genes for phylogenetic analysis of major serotypes of *A. actinomycetemcomitans*. The results are in agreement with previous studies that used limited numbers of genetic markers or genome alignment for phylogenetic analysis of *A. actinomycetemcomitans* [16], [17], [18], [19]. The data suggest a major evolutionary division between the group of serotypes a, d, e and f strains and the group of serotypes b and c strains.

The heterogeneity within the serotype e strains of *A. actinomycetemcomitans* found in our study has been suggested previously. Dogan et al. [20] distinguished three groups of serotype e strains by AP-PCR typing and by sugar fermentation profiling or two genotypes based on the restriction analysis of the *apaH* amplification product. Also, two groups of serotype e strains of *A. actinomycetemcomitans* were identified (e and e′) based on 16S rRNA gene sequence similarity and amplified fragment length polymorphism typing [21]. In this study, strain SC1083 could be assigned to the serotype e′ and the strain SCC393 to the conventional serotype e group based on the signature sequences of the 16S rRNA genes described by Reijden et al. [21]. It is tempting to speculate that each subtype of serotype e strains may represent a distinct clonal lineage. Additional serotype e strains of both subtypes should be examined to verify this possibility.

### Size of the core genome, flexible gene pool, and pangenome

**Figure 2:**
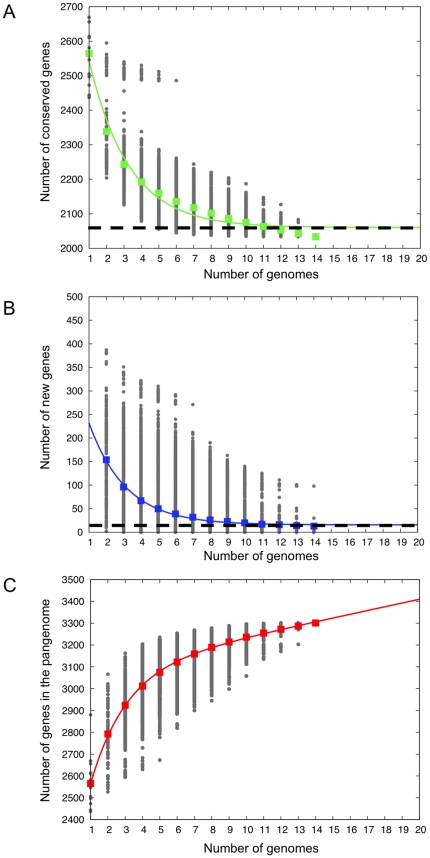
Analysis of pangenome and its components in *A. actinomycetemcomitans*. Analysis of the size of the core genome (A) and whether the pangenome is open or closed (B and C). The number of common genes (A), new genes (B) and size of pangenome (C) are plotted as a function of the number of *n* of strains sequentially added. Circles show all different strain combinations for each *n*. Squares are the averages for each *n*. The continuous curve in A and B represents the least-squares fit of an exponential decay function. The extrapolated *A. actinomycetemcomitans* core genome size and average number of new genes are shown as dashed lines in top and middle plots respectively. Based on the curve, the core genome size is estimated to be about 2,060 genes. For every additional strain up to 16 new genes can be added to the open pangenome.

Counting protein-coding genes only, the size of the pangenome (the total unique genes of the 14 strains) was 3,301 genes, which included 2,034 core genes (genes found in all strains) and 1,267 flexible genes (present in some but not all 14 strains). The data was used to further assess the size of the core gene pool and the pangenome by mathematical modeling (Figure 2). The core gene pool approached 2,060 genes and the addition of new strains for analysis beyond the 14 examined strains was not expected to significantly affect its size (Figure 2A). The pangenome of *A. actinomycetemcomitans* was found to be open-ended (Figure 2C). Sixteen new genes are estimated to be added to the pangenome for each additional strain sequenced (Figure 2B).

**Figure 3:**
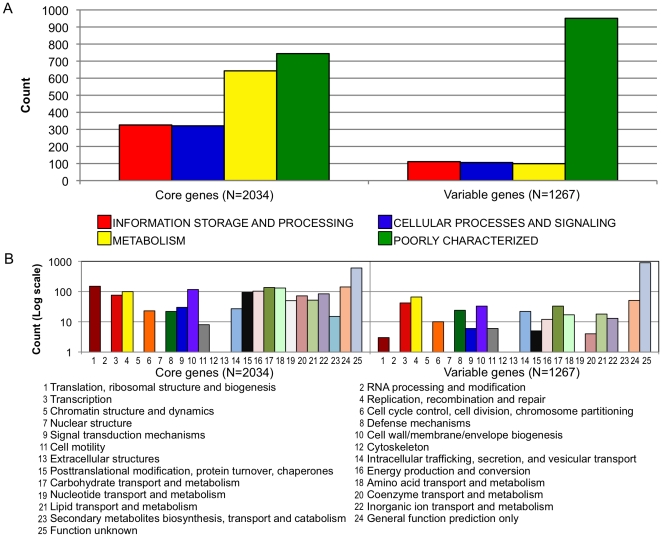
Classification of core and flexible genes in *A. actinomycetemcomitans*. Genes were classified by the COG super-functional category (A) and the COG functional category (B). As expected, the core genome is better represented by genes that provide the essential cell function for the bacterium whereas the vast majority of the variable genome is made up of poorly characterized genes. Poorly characterized genes also represented about one third of the core genome. This suggests that more studies are still needed to understand the basic biology of this bacterial species.

The distribution patterns of the flexible genes (subgrouped based on the numbers of strains sharing the genes) in individual strains are illustrated in Figure S2. Within each strain 16.7–29.4% of the genome belonged to the flexible gene pool. Figure 3 shows the functional classification of core and flexible genes by COG super-functional (Figure 3A) or functional category (Figure 3B). As expected, the vast majority of genes making up the core genome belonged to the groups of housekeeping functions. As is common in most bacteria, about one-fourth of the shared genes were assigned to the category of poorly characterized proteins, suggesting that many aspects of basic *A. actinomycetemcomitans* biology still need to be explored. Genes associated with housekeeping functions were also found within the flexible gene pool but less well-represented there, whereas poorly characterized genes comprised the majority of the flexible pool; 951 out of 1,267 (75%) flexible genes were classified as poorly characterized (Figure 3A). One hundred and sixty-seven of the remaining 316 flexible genes (53%) are associated with mobile and extrachromosomal elements.

In this study the core genome of *A. actinomycetemcomitans* accounts for 70.6% to 83.3% of any single genome. The sizes of the core genome or the flexible gene pool of *A. actinomycetemcomitans* are not unusual in comparison to other bacterial species. Welch et al. [22] examined three strains of *Escherichia coli* and showed that less than 40% of the genomes were shared. Presumably the size of the core genome will be even smaller with additional *E. coli* strains included for comparison. In a study of 12 *Procholorococcus* isolates, the size of the core genome approached 1,250 genes, or from 40% to 67% of the genomes of individual isolates. Among *Streptococcus agalactiae* strains 80% of the genome of any strain was shared [23].

### Genome comparison among *A. actinomycetemcomitans* strains

**Figure 4:**
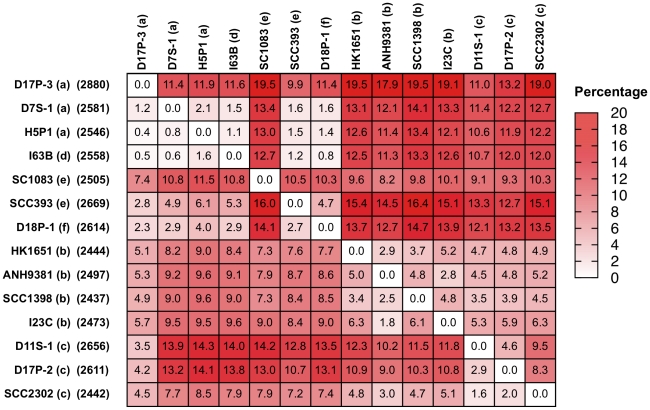
Pair-wise genomic comparisons among *A. actinomycetemcomitans* strains. A heat map of the genome variation among 14 *A. actinomycetemcomitans* strains is shown. The numbers in the box show the % of protein-coding genes found in one genome (left) but not another. The data are organized by serotypes; a (D17P3, D7S-1, and H5P-1), d (I63B), e (SC1083), e (SCC393), f (D18P1), b (HK1651, ANH9381, SCC1398, I23C), c (D11S-1, D17P2, SCC2302). The patterns of genome similarity mirror those found in phylogenetic analysis by 16S rRNA gene sequences or 25 housekeeping genes shown in Figures 1 and 2. The numbers in parenthesis on the left of the map indicate the numbers of protein-coding genes.

**Figure 5:**
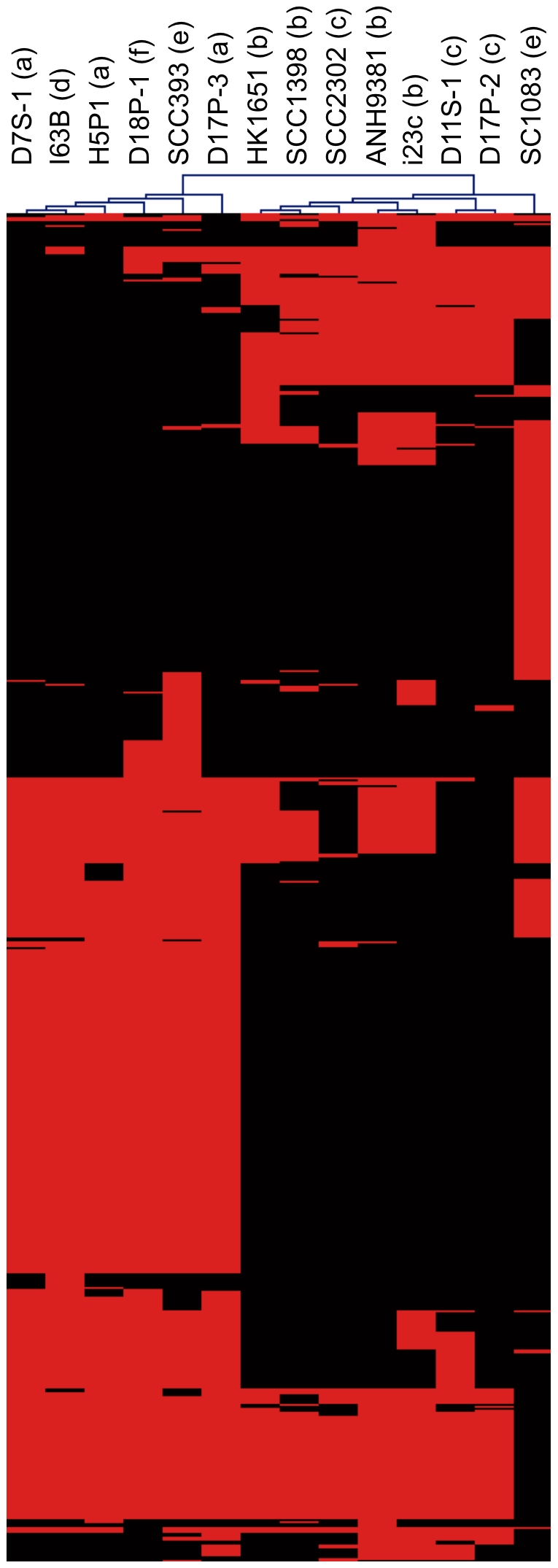
Two-way hierarchical clustering of the variable gene profiles of *A. actinomycetemcomitans* strains. Columns represent *A. actinomycetemcomitans* genomes. Rows represent genes. Red and black color cells represent presence or absence of genes in a particular genome respectively. Clustering result shows a similar grouping of *A. actinomycetemcomitans* strains as phylogenetic analysis where strains from serotype a, d, e and f (excluding strain SC1083) form a group and strains from serotype b and c form another group. Image truncated for brevity.

Figure 4 provides a summary of strain-to-strain comparisons of protein-coding genes of the genomes. Depending on the pair of strains compared and the direction of the comparison, the percentage of genes present in one genome but not in another ranged from 0.4% to 19.5%. Two groups of strains were recognized based on the similarity matrix. One group was formed by the strains of serotypes a, d, e (except strain SC1083) and f. The other group was formed by the strains of serotypes b and c. The serotype e strain SC1083 did not seem to follow the pattern. Two-way hierarchical clustering analysis of the flexible genes also illustrated the same patterns of two major groups and the distinctiveness of strain SC1083 (Figure 5). The segregation in the pattern of genomic variation among strains mirrored that found in phylogenetic analysis using housekeeping genes (Figure 1).

The natural population of *A. actinomycetemcomitans* is clonal, with each serotype representing a distinct clone lineage [18]. However, the extent of the genomic variation within individual clonal lineages has not previously been known. In this study multiple strains of each of the serotypes a, b, c and e were available for comparison. The strain-to-strain variations in genome content were 0.4–11.9% for serotype a strains, 10.5–16% for serotype e strains, 1.8–6.3% for serotype b strains, and 1.6–9.5% for serotype c strains (Figure 4). The variations between serotypes a and e were 1.2–19.5%, and between serotypes b and c were 3.0–12.3%. Therefore, the genomic variation within each serotype in some instances may be as great as that between serotypes, suggesting that phenotypic variation (including virulence potentials) of *A. actinomycetemcomitans* strains cannot be expected to comply with the serotype group.

Strain HK1651 belongs to the JP2 clone, which comprises strains marked by the 530-bp deletion of the leukotoxin promoter and are clonally identical based on multilocus enzyme analysis [24], [25], [26], [27]. The term “non-JP2 clones” has been loosely applied to all serotypes of *A. actinomycetemcomitans* strains that do not have the characteristic 530-bp deletion [7]. The present study has clearly demonstrated significant variation of the genome content among strains of different serotypes. Comparison of genetically restricted JP2 clone to genetically diverse “non-JP2 clones” is not informative. Here we propose that the terms “JP2” and “non-JP2” should apply only to serotype b strains for a meaningful comparison of the closely related group of bacteria with or without the particular genetic marker.

### Identification of genomic islands among *A. actinomycetemcomitans* strains

A total of 171 genomic islands were found among the 14 *A. actinomycetemcomitans* strains (Table 1). Some of these islands resembled phage or plasmid sequences. Cumulatively 777 strain-specific genes were found on these islands and represented 61% of the flexible gene pool and showed similar COG distribution pattern with the flexible gene pool.

On average 26.7% of the variable genes found on the genomic islands in each genome (in comparison to 8.5% of the core genes) displayed atypical nucleotide composition of at least two standard deviations from the mean values in one of three base composition values (%G+C, genome signature and codon bias) [28] (see Table S3 for the list of genomic islands, genes and base composition analysis). Evidently nearly all genomic islands identified in this study were novel. The functions of these islands remain to be elucidated.

The occurrence of each genomic island among the 14 strains is provided in Table S4. On average each of the 171 islands was found in 3.1 strains. Only one island was found to occur in more than 10 strains. While the presence of the “strain-specific regions” in the genomes can be interpreted as genomic islands, or alternatively as deletions (in the strains that do not have the “strain-specific regions”), the latter could be ruled out based on the distribution patterns of these islands among strains. It is less likely that a majority of the *A. actinomycetemcomitans* strains would have independently suffered a deletion in identical genomic regions.

Genomic islands may be identified by base composition analysis as well as by phylogenetic approaches [29]. No single method is considered optimal or universally accepted as the best approach for identification of genomic islands. For example, base composition analysis may not identify ancient genomic islands due to amelioration of the horizontally acquired genes over time [30]. In this study, comparative genomics has the advantage of directly identifying strain-specific DNA regions as putative genomic islands. Prior to this study, there was limited information about the genomic islands of *A. actinomycetemcomitans*. Eight genomic islands have been identified in strain HK1651 (http://www.oralgen.lanl.gov/_index.html), including four islands also identified independently in this study. Among the remaining four islands three were species-specific genomic islands (ie, present in all examined strains; the islands of tight adherence gene cluster, leukotoxin gene cluster and LOS biosynthesis enzyme) and one was too small (cytolethal distending toxin gene cluster, <5 kb) to be categorized as a genomic island in this study. Finally, this study also identified 32 DNA regions that have many features of genomic islands (Table S5). For example, each has one or more flexible genes with atypical base compositions characterized by 1 standard deviation from the mean values, and carries mobile elements and genes encoding phage proteins.

### Variable presence of virulence determinants in *A. actinomycetemcomitans* strains

It is known that the ∼2.4 kb *cdtABC* operon of *A. actinomycetemcomitans* resides on a genomic island [31]. In this study a homologous ∼14 kb *cdtABC*-carrying genomic island (here designated as *cdt*-island) was identified in five *A. actinomycetemcomitans* strains based on its presence in a finished genome or its clear delineation within single contigs of the unfinished genomes. These strains were serotype a strains D7S-1 and D17P3, serotype d strain I63B, serotype e strain SCC393, and serotype f strain D18P1. The presence of the *cdt*-island in serotype a strain H5P1 was undefined because it was distributed in three different contigs. While the *cdtABC* operon was also found in the serotype b and c strains in this study, no apparent genomic island was associated with the operon. Instead, a ∼5 kb homologous region that carried the ∼2.4 kb *cdt* operon was found among the serotype b and serotype c strains.

The ∼14 kb *cdt*-islands identified in this study were different from a previously reported genomic island (designated GIY4-1) of *cdtABC* operon [31] in a serotype b strain Y4. Sequence analyses of these two types of genomic islands showed a ∼4 kb region of sequence homology that included the ∼2.5 kb *cdtABC* and its upstream 1.3 kb region.

**Figure 6:**
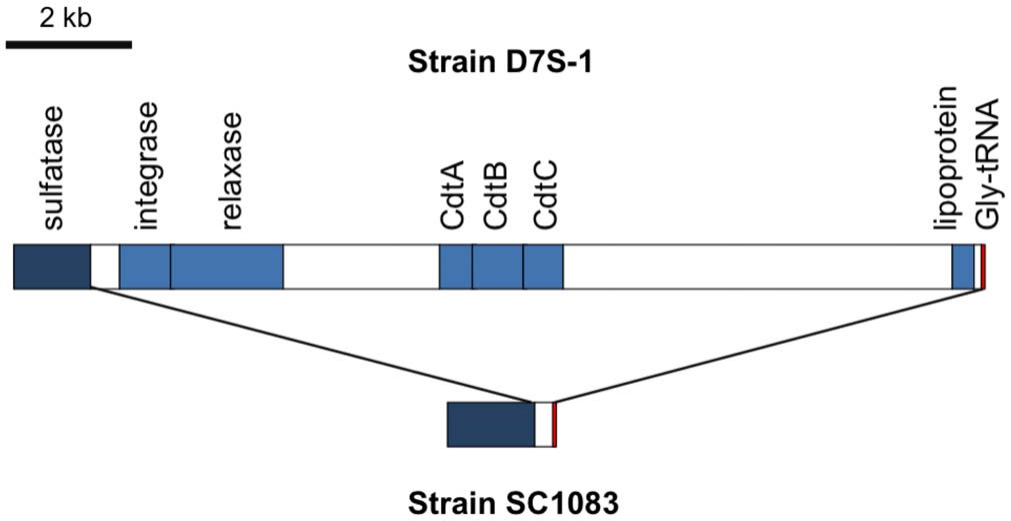
Genetic comparison between *A. actinomycetemcomitans* strains with or without the *cdt*-island. Comparison of the genetic locus of the *cdt*-island in strain D7S-1 and the comparable locus in strain SC1083. The homologous sulfatase and Gly-rRNA between strains are colored in dark blue and red, respectively. Several genes encoding hypothetical proteins and other proteins in the *cdt*-island of strain D7S-1 are not indicated in the map (see Table S3 for a full list of genes on the island). The comparison shows that strain SC1083 is missing the ∼14 kb *cdt*-island.

Interestingly, the ∼14 kb *cdt*-island (and the *cdtABC*) was either not acquired by strain SC1083 or was deleted from the strain during its evolution. The evidence is shown in the comparison of the locus between SC1083 and D7S-1 (Figure 6) (see Figure S3 for sequence confirmation). The flanking regions of the *cdt*-island in D7S-1 were found as a single contiguous DNA region in SC1083 without the island. Notably a Gly-tRNA gene was identified in both strains. Genomic islands are often associated with tRNA genes, which may serve as a preferred integration sites [32], [33]. The results also suggest that the *cdt-*island was acquired en bloc in some *A. actinomycetemcomitans* strains.

A putative virulence factor CagE [34], [35] (designated as p-cluster 03282 in this study) was found only in two serotype b strains HK1651 (JP2 clone) and SCC2302. The significance of the presence or absence of CagE to the virulence of *A. actinomycetemcomitans* strains remains to be examined.

*A. actinomycetemcomitans* strains are known to exhibit variation in natural competence for DNA uptake. Naturally competent *A. actinomycetemcomitans* strains were identified in serotypes a, d and e but not in serotypes b or c [36]. One possible reason for the lack of detectable natural competence is a common insertional inactivation of a *comM* gene in strains of serotypes b and c [37]. In this study we found that all serotype a strains (D7S-1, D17P3, and H5P1) and the serotype e strain SCC393 had an intact full-length *comM* gene. In contrast, the *comM* of serotype b and c strains (HK1651, ANH9381, SCC1398, SCC2302, D11S-1, D17P-2), and the serotype e strain SC1083 and serotype f strain D18P-1, were apparently inactivated by the insertion of a genomic island (∼14–25 kb) that showed significant homology among strains. The results again suggest that the insertional inactivation of *comM* gene occurred in some clonal lineages of *A. actinomycetemcomitans*. The significance of such insertional event in the evolution of *A. actinomycetemcomitans* remains to be investigated.

### Genome comparison between *A. actinomycetemcomitans* and *Aggregatibacter aphrophilus*

*A. aphrophilus* is closely related to *A. actinomycetemcomitans*. While both species belong to the HACEK group that is often associated with nonoral infections, the former is not considered a periodontal pathogen. A preliminary comparative genomic analysis was performed to examine the presence of known virulence determinants of *A. actinomycetemcomitans* in the genomes of *A. aphrophilus*. The homologs of fimbrial gene cluster [38], [39], *aae* [40] and *emaA* [41] were found in both *A. aphrophilus* and *A. actinomycetemcomitans*. However, leukotoxin operon, *cdtABC* operon or *kat* (for catalase) [42] were found in *A. actinomycetemcomitans* (except in strain SC1083 which does not have *cdtABC*) but not *A. aphrophilus*. It is premature to conclude that the presence or absence of these virulence determinants accounts for the distinctions in their periodontal virulence potentials. Nevertheless, comparative genomics of closely related oral haemophili may be a valid approach to examine the etiopathogenesis of *A. actinomycetemcomitans*-associated periodontal disease.

Finally, the strain-dependent genomic differences have implications in applications such as microarray design for *A. actinomycetemcomitans*. With such genomic variation, a microarray created based on the genome content of a single strain will not provide comprehensive information if applied to a strain, or strains with significant differences in genome content. We have applied the information obtained in this study and designed a pangenome microarray for *A. actinomycetemcomitans* (accessible through https://earray.chem.agilent.com/earray/ under “Published Designs” by species “Na”. or alternatively, https://earray.chem.agilent.com/earray/showPublishPageForLogin.do?action=showPublishResultsForLoginPage&species=Na under “aa_array.100315”), and successfully tested on clinical isolates for genome content analysis and for transcriptome analysis (manuscripts in preparation).

## Materials and Methods

### Bacteria and genome sequences

Eleven clinical strains of *A. actinomycetemcomitans* (2, 3, 2, 1, 2, 1 strains of serotypes a, b, c, d, e, and f, respectively) were selected for whole genome sequencing. These strains were cultivated from subgingival plaque of 11 noncohabiting individuals with different periodontal disease diagnoses. All strains were verified as *A. actinomycetemcomitans* by a 16S rDNA-based PCR assay and their serotypes were determined as described previously [36], [43], [44], [45]. *A. aphrophilus* ATCC 33389 (purchased from ATCC) was also sequenced. The genome sequencing was performed using 454 pyrosequencing technology [46] and run on a Genome Sequencer FLX Instrument (Software 1.0.53) following the manufacturer's instruction (Hoffmann-La Roche Ltd). The raw data were assembled using the Newbler Assembler Software (Genome Sequencer 20, Version 1.0.53), with default parameters. The Newbler Metrics of 454 sequencing and Genbank accession numbers are provided in Table S1. The Genbank accession numbers are; ADOA00000000 (D17P-3), AEJK00000000 (H5P1), ADOC00000000 (ANH9381), AEJQ00000000 (i23C), AEJP00000000 (SCC1398), AEJR00000000 (SCC2302), ADOB00000000 (D17P-2), AEJL00000000 (I63B), AEJN00000000 (SCC393), AEJM00000000 (SC1083), AEJO00000000 (D18P-1) and AEWB00000000 (*A. aphrophilus* ATCC 33389).

### Gene prediction and annotation

In addition to the 11 *A. actinomycetemcomitans* strains sequenced in this study, the genome sequences of three previously sequenced *A. actinomycetemcomitans* strains [14], [15] (http://www.genome.ou.edu/act.html), *A. aphrophilus* strain NJ 8700 [47] and ATCC 33389 were included in the analysis. The sequences were processed by our in-house pipeline for gene prediction and annotation as described previously [14], [15]. Briefly, Glimmer3 [48], Exonerate [49], and tRNAscan SE tools [50] were used to predict protein-, rRNA- and tRNA-coding genes, respectively. All protein-coding genes were annotated by BLAST searching against Genbank non-redundant protein sequence database (Blastp with E-Value cutoff of 1e-6). The description of the best BLAST hit was then used as an annotation for that gene. Each protein sequence was also BLAST searched against the Clusters of Orthologous Groups of proteins (COGs) database [51] and a COG identification number was assigned to each gene if the best BlastP hit exhibits at least 80% sequence coverage in both query and hit sequences and at least 30% protein sequence identity. Because our genome sequence data were produced using the 454 pyrosequencing technology, which tends to produce insertion/deletion (indel) nucleotide polymorphism in homopolymer stretches, protein-coding genes were further analyzed to identify putative frameshift mutations using BLAST Extend-Repraze (http://ber.sourceforge.net/). Additionally, BLAST hits of each protein-coding gene were analyzed to identify cases where indels in homopolymer stretch have caused a potentially correct stop codon to be out of frame and created a predicted gene that was a fusion between two genes, which was then corrected manually.

### Identification and grouping of homologous genes

In order to analyze genes across multiple genomes, we created a method to identify and group genes that were homologous to one another. Initially, each gene from all genomes was treated as an independent gene cluster (defined as a collection of homologous genes) of one gene. The number of gene clusters at this initial step was therefore equal to the total number of predicted genes from all genomes. A representative gene sequence of each cluster, which we defined as the longest member, was then used to search all genomes to identify other homologous genes. Four different modes of sequence comparison were employed for identification of homologous genes (ie, belonging to the same gene clusters): (i) comparison of a representative gene DNA sequence to all gene DNA sequences database using BlastN, (ii) comparison of a representative gene DNA sequence to all genome sequences database using BlastN, (iii) comparison of a representative Protein sequence to all gene Protein sequences database using BlastP, (iv) comparison of a representative Protein sequence to all translated genome Protein sequences database using TBlastN.

For i) and iii), significant BLAST hits that had e-values of less than 1e-6 and exhibited at least 50% sequence identity and 50% sequence coverage between query and hit sequences were considered homologous to the query representative sequence. Similarly, for ii) and iv), significant BLAST hits that showed at least 50% sequence identity and 50% sequence coverage between the query representative sequence and the sequence in the genome were considered homologous. Any genes found within these homologous genomic regions were automatically considered as homologs to the query representative sequence. This representative gene versus genome sequence comparisons allowed us to identify and group genes that may have been predicted inconsistently in different genomes due to factors such as differences in sequence quality (especially near the ends of a contig), frameshift mutations, and the incomplete nature of genome sequences. Specifically, these factors could cause a gene prediction program to call fragmented genes instead of a full length version or a significantly shorter gene relative to homologs found in other genomes. Genes that showed homology to the representative sequence by any of the four comparison modes were then removed from their clusters and added to the cluster that was the origin of the representative sequence. For ii) and iv), if a homologous genomic region was found but no gene was predicted in that region, a note for that particular genome was added to the cluster. This allowed us to differentiate a true missing gene from an artifact in gene prediction result. This homologous gene grouping process was iterated for all non-empty clusters until no change was found in the gene cluster membership after a whole round of comparison had been carried out. Finally, manual inspection was performed to further group genes that are obviously homologous but were not grouped together by our criteria.

### Phylogenetic analysis

Twenty-five housekeeping genes, most of which coded for 30S and 50S ribosomal subunit proteins (see Table S2), common to 14 *A. actinomycetemcomitans* strains, two *A. aphrophilus* strains, and *Haemophilus influenzae* 028NP, *H. influenzae* Rd, *H. influenzae* PittGG, *H. influenzae* PittEE, *Haemophilus somnus* 129PT, *H. somnus* 2336, *Haemophilus ducreyi* 35000HP, and *Mannheimia haemolytica* PHL213, were selected for analysis. The nucleotide sequences of individual genes were aligned using ClustalW version 2 with default parameters [52]. Gaps were removed from all alignment results, which in turn were concatenated to produce a single alignment (a total length of 17,840 bp) in PHYLIP format. PHYLIP program version 3.6 was used to construct the tree using the maximum likelihood method (dnaml) (http://evolution.genetics.washington.edu/phylip/getme.html) [53]. A bootstrap analysis was carried out to test the reliability of the tree. Finally the DRAWGRAM software (part of the PHYLIP package) was used to draw the tree.

### Identification of genomic islands

Initially, three different base composition analyses: namely, G+C content, dinucleotide bias (also known as genomic signature), and codon bias were performed for predicted genes in each genome. The methods established by Karlin [47] were used to calculate the dinucleotide and codon bias values for each coding sequence. The mean and standard deviation for each of these base composition values were calculated. Genomic islands of at least 5 kb in length were then identified by a step-wise process. First, regions of at least 1 kb containing a gene not shared by all strains were identified. The variable regions were then merged if they were within 5 kb of each other. Finally, the resulting regions of at least 5 kb that contain at least one gene that displays atypical base composition (as characterized by at least 2 standard deviations in any of the three base composition values) were considered as genomic islands.

### Estimation of the size of the core genome and the pangenome of 14 *A. actinomycetemcomitans* strains

The core genome and the pangenome were estimated based on a method described by Tettelin et al. [23]. In brief, all possible combinations of sequential inclusion of up to 14 strains were simulated and a regression analysis was used to fit an exponential decaying function to the amount of conserved genes and of strain-specific genes. This allowed us to estimate and extrapolate the size of the core genome and the pangenome if additional *A. actinomycetemcomitans* strains were available for genome sequencing and analysis.

## Supporting Information

Figure S1 **DNA sequence similarity within gene clusters.** Histogram shows the distribution of percent DNA sequence similarity between genes and their corresponding gene cluster representatives. Seventy-six percent of the genes showed 95–100% sequence similarity to their cluster representative sequences. No genes showed less than 75% DNA sequence similarity to their cluster representatives.
(DOCX)

Figure S2 **Distribution patterns of core and flexible genes in** ***A. actinomycetemcomitans*** **strains.** This figure shows cumulative percentage of genes (y-axis) that are found in different number of the 14 *A. actinomycetemcomitans* genomes studied. The genes are color coded based on the numbers of genomes that share the genes (see right side of the figure for the color coding). This analysis shows that about 20% or less of the genes in each *A. actinomycetemcomitans* genome constitute a variable gene pool.
(DOCX)

Figure S3 **Sequence confirmation of the deletion of** ***cdt*** **-island in strain SC1083.** See Figure 6 for the genetic map. Two primers (underlined) annealing to sulfatase and Gly-tRNA were used to amplify a 660 bp DNA fragment for sequencing. The result is identical to the sequencing information of SC1083 from pyrosequencing. The intergenic region is indicated with lower case letters, and the ends of the flanking genes indicated by the upper case letters.
(DOCX)

Table S1 Newbler Metrics of 454 sequencing of 11 *A. actinomycetemcomitans* strains and the *A. aphrophilus* strain ATCC 33389.
(DOCX)

Table S2 List of genes of *A. actinomycetemcomitans* for phylogenetic analysis.
(DOCX)

Table S3 List of genomic islands and their gene content in *A. actinomycetemcomitans* strains.
(XLSX)

Table S4 The distribution of individual genomic islands among *A. actinomycetemcomitans* strains.
(XLSX)

Table S5 Additional DNA regions with atypical base composition among *A. actinomycetemcomitans* strains.
(XLSX)
