# Supplementary figures and images for: Identification of the Pangenome and Its Components in 14 Distinct Aggregatibacter actinomycetemcomitans Strains by Comparative Genomic Analysis
Source: PLoS One. 2011 Jul 19;6(7):e22420. doi: 10.1371/journal.pone.0022420 (PMC3139650; doi:10.1371/journal.pone.0022420)

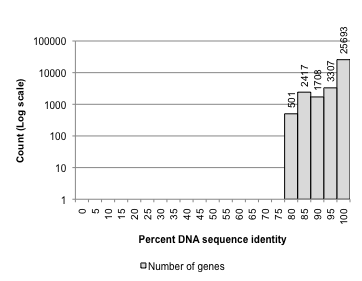


**Figure S1.**

Supplement: Figure S1 — DNA sequence similarity within gene clusters. Histogram shows the distribution of percent DNA sequence similarity between genes and their corresponding gene cluster representatives. Seventy-six percent of the genes showed 95–100% sequence similarity to their cluster representative sequences. No genes showed less than 75% DNA sequence similarity to their cluster representatives. (DOCX) [file pone.0022420.s001.docx]

**
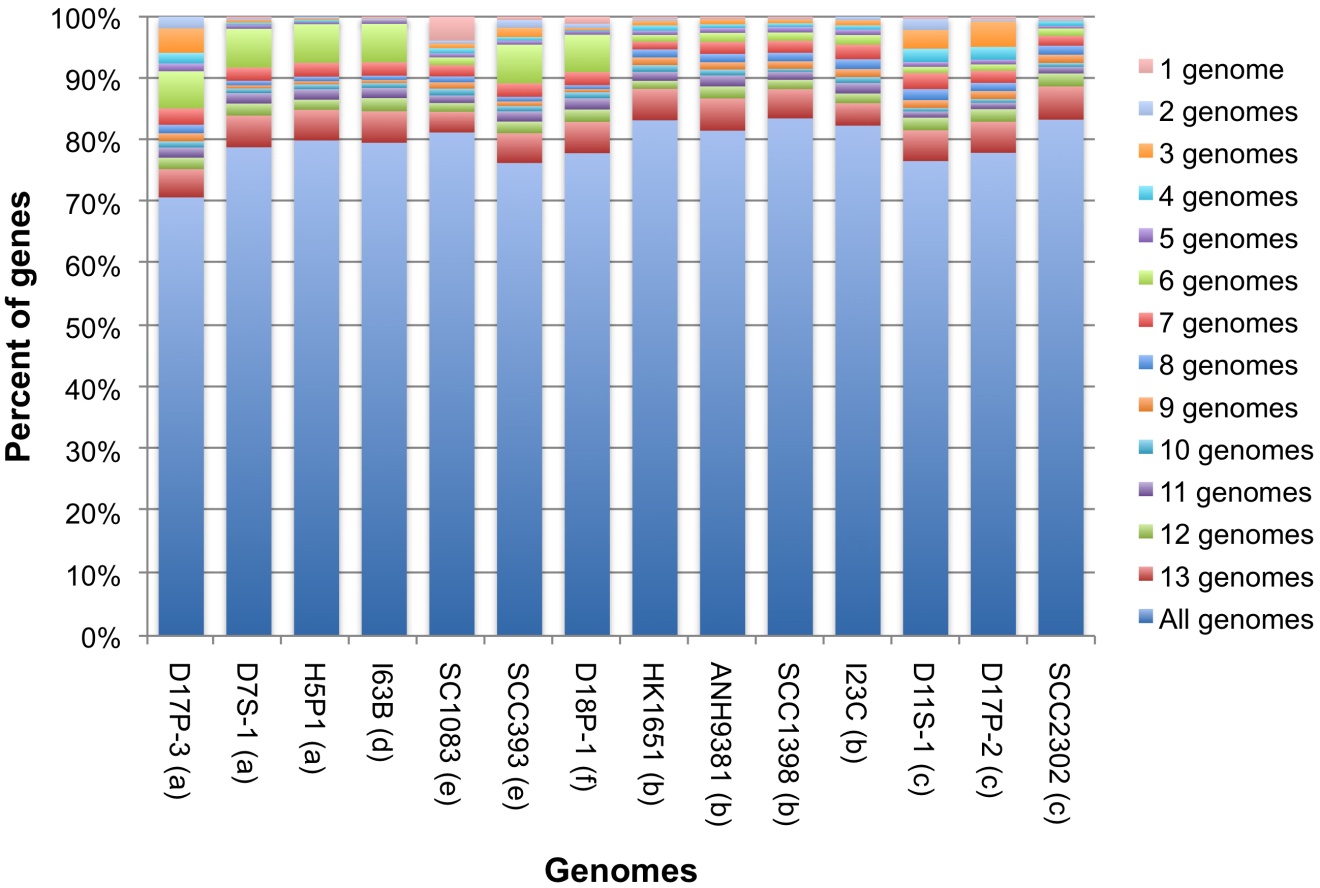
**

**Figure S2.**

Supplement: Figure S2 — Distribution patterns of core and flexible genes in A. actinomycetemcomitans strains. This figure shows cumulative percentage of genes (y-axis) that are found in different number of the 14 A. actinomycetemcomitans genomes studied. The genes are color coded based on the numbers of genomes that share the genes (see right side of the figure for the color coding). This analysis shows that about 20% or less of the genes in each A. actinomycetemcomitans genome constitute a variable gene pool. (DOCX) [file pone.0022420.s002.docx]
