# Supplementary material for: Identification of the Pangenome and Its Components in 14 Distinct Aggregatibacter actinomycetemcomitans Strains by Comparative Genomic Analysis
Source: PLoS One. 2011 Jul 19;6(7):e22420. doi: 10.1371/journal.pone.0022420 (PMC3139650; doi:10.1371/journal.pone.0022420)
Supplement: Figure S3 — Sequence confirmation of the deletion of cdt -island in strain SC1083. See Figure 6 for the genetic map. Two primers (underlined) annealing to sulfatase and Gly-tRNA were used to amplify a 660 bp DNA fragment for sequencing. The result is identical to the sequencing information of SC1083 from pyrosequencing. The intergenic region is indicated with lower case letters, and the ends of the flanking genes indicated by the upper case letters. (DOCX) [file pone.0022420.s003.docx]

TTGAGTCGGTGGGGTTTCCGGTATTGCTTGTTTTCTTAGTCTATATAAAATATATCCGGATAAAATAAATAAGAATGGCAGTAAAAATTGGCTAAATTTTAATTTTCCTAAAAATTCAAAAGATTCTTCCGGGTTGGTTTCAAAAAAaGCAGCAACAACTCCGGAGTTTAAGTTTCCATAAGATAAATATATAGGCTGATAAATAGATAACGTTATGATAACGAATGTAATAAGGCTCCAGAATAATTTCTGATTTAGTGAGAATATTAAATAATAGATAATGTATATTACAAACATGCCATAATAAATAGTATTCTCAGGTGATATAATAAGCGTAATAAATGAGAAAAAGAGTAGCCAAATCCAAAAGATTCGGCTATTTAGATAATTAAGAAATTTTTTAAACATaaattggttctttaggtttaagactgaagataacagaaaagacaaagtaagagtgctaattttatacctaataacttataaactcaatagcattagggatatgccaatgtaaagataagttaaagatttgtcagtttgtattgaaaaacactgggaattctgaccgcacttttcagtctcaaggtcgcgcacagaataaaaaatccctcgctgaaaggcgagggattgtaaattTGGAAGCGGGAAACGAGGCTCGAAC

**Figure S3**
