# Supplementary material for: Identification of the Pangenome and Its Components in 14 Distinct Aggregatibacter actinomycetemcomitans Strains by Comparative Genomic Analysis
Source: PLoS One. 2011 Jul 19;6(7):e22420. doi: 10.1371/journal.pone.0022420 (PMC3139650; doi:10.1371/journal.pone.0022420)
Supplement: Table S1 — Newbler Metrics of 454 sequencing of 11 A. actinomycetemcomitans strains and the A. aphrophilus strain ATCC 33389. (DOCX) [file pone.0022420.s004.docx]

**Table S1.** Newbler Metrics of 454 sequencing of 11 *A. actinomycetemcomitans (Aa)* strains and the *A. aphrophilus* strain ATCC33389

| Species | *Aa* | *Aa* | *Aa* | *Aa* | *Aa* | *Aa* | *Aa* | *Aa* | *Aa* | *Aa* | *Aa* | *A. aphrophilus* |
| --- | --- | --- | --- | --- | --- | --- | --- | --- | --- | --- | --- | --- |
| Strain | D17P-3 | H5P1 | Anh9381 | i23c | SCC1398 | SCC2302 | D17P-2 | I63B | SCC393 | SC1083 | D18P-1 | ATCC33389 |
| Coverage | 25X | 25X | 16X | 16X | 29X | 30X | 28X | 16X | 19X | 36X | 43X | 16X |
| No. of Large Contigs (#bases) | 267 (2,384,518) | 296 (2,151,174) | 102 (2,112,345) | 400 (2,020,423) | 137 (2,167,288) | 46 (2,037,488) | 62 (2,173,675) | 174 (2,175,418) | 200 (2,222,051) | 65 (2,164,541) | 91 (2,237,538) | 45 (2,265,789) |
| Avg Contig Size (bp) | 8,930 | 7,267 | 20,709 | 5,051 | 15,829 | 44,293 | 35,059 | 12,502 | 11,110 | 33,300 | 24,588 | 50,350 |
| Median Contig Size (bp) | 21,466 | 13,510 | 33,704 | 7,958 | 4,592 | 102,488 | 84,363 | 21,401 | 18,019 | 91,214 | 63,757 | 144,921 |
| Q39 Bases* | 15,470 (0.65%) | 18,939 (0.88%) | 17,627 (0.83%) | 33,125 (1.64%) | 7,344 (0.34%) | 11,367 (0.56%) | 7,363 (0.34%) | 16,265 (0.75%) | 21,893 (0.99%) | 2,538 (0.12%) | 752 (0.03%) | 3,214 (0.14%) |

*Bases with a quality score of less than or equal to 39 which is equivalent to an error rate of approximately 1 in 10,000 bases
