# Supplementary material for: Identification of the Pangenome and Its Components in 14 Distinct Aggregatibacter actinomycetemcomitans Strains by Comparative Genomic Analysis
Source: PLoS One. 2011 Jul 19;6(7):e22420. doi: 10.1371/journal.pone.0022420 (PMC3139650; doi:10.1371/journal.pone.0022420)
Supplement: Table S2 — List of genes of A. actinomycetemcomitans for phylogenetic analysis. (DOCX) [file pone.0022420.s005.docx]

**Table S2.** List of genes for phylogenetic analysis

|  | **Gene Name** | **Gene Product** | **D7S-1 location** | **HK1651 location** | **D11S-1 location** |
| --- | --- | --- | --- | --- | --- |
| 1 | rpoB | DNA-directed RNA polymerase subunit beta | 1,598,699 | 234,946 | 48,950 |
| 2 | infB | initiation factor IF2-1 | 359,473 | 1,294,850 | 1,113,935 |
| 3 | groEL | chaperone GroEL | 1,197,747 | 868,714 | 538,258 |
| 4 | pgk | phosphoglycerate kinase | 828,007 | 965,425 | 327,948 |
| 5 | truB | tRNA-pseudouridine synthase I | 356,063 | 1,305,238 | 1,117,342 |
| 6 | rpsC | ribosomal protein S3 | 1,705,029 | 245,691 | 59,693 |
| 7 | rplA | ribosomal protein L1 | 1,600,929 | 232,710 | 46,714 |
| 8 | rplD | ribosomal protein L4 | 1,702,625 | 248,095 | 62,097 |
| 9 | rplF | ribosomal protein L6 | 760,530 | 840,993 | 510,240 |
| 10 | rplJ | ribosomal protein L10 | 1,599,884 | 233,761 | 47,765 |
| 11 | rplO | ribosomal protein L15 | 758,924 | 842,599 | 511,846 |
| 12 | rplK | ribosomal protein L11 | 1,601,362 | 232,277 | 46,281 |
| 13 | rplP | ribosomal protein L16 | 1,705,750 | 244,970 | 58,972 |
| 14 | rpsI | ribosomal protein S9 | 119,882 | 1,538,542 | 1,347,282 |
| 15 | rpsH | ribosomal protein S8 | 760,938 | 840,585 | 509,832 |
| 16 | rplQ | ribosomal protein L17 | 754,412 | 847,114 | 516,361 |
| 17 | rpsF | ribosomal protein S6 | 812,011 | 937,044 | 356,337 |
| 18 | rplW | ribosomal protein L23 | 1,703,224 | 247,496 | 61,498 |
| 19 | rpsT | ribosomal protein S20 | 1,726,328 | 2,059,088 | 1,849,156 |
| 20 | rpsQ | ribosomal protein S17 | 1,706,351 | 244,369 | 58,371 |
| 21 | rpsP | ribosomal protein S16 | 797,955 | 922,983 | 370,398 |
| 22 | rpsR | ribosomal protein S18 | 811,308 | 936,341 | 357,040 |
| 23 | rpsD | ribosomal protein S4 | 756,092 | 845,434 | 514,681 |
| 24 | rplR | ribosomal protein L18 | 759,908 | 841,540 | 510,835 |
| 25 | rpsS | ribosomal protein S19 | 1,704,523 | 246,326 | 60,199 |
